# An Update in Epigenetics in Metabolic-Associated Fatty Liver Disease

**DOI:** 10.3389/fmed.2021.770504

**Published:** 2022-01-11

**Authors:** J. Samael Rodríguez-Sanabria, Rebeca Escutia-Gutiérrez, Rebeca Rosas-Campos, Juan S. Armendáriz-Borunda, Ana Sandoval-Rodríguez

**Affiliations:** ^1^Department of Molecular Biology and Genomics, Institute for Molecular Biology in Medicine and Gene Therapy, CUCS, University of Guadalajara, Guadalajara, Mexico; ^2^School of Medicine and Health Sciences, Tecnologico de Monterrey, Campus Guadalajara, Zapopan, Mexico

**Keywords:** MAFLD, NASH, DNA methylation, histone modification, miRNAs

## Abstract

Metabolic-associated fatty liver disease (MAFLD) is characterized by hepatic steatosis accompanied by one of three features: overweight or obesity, T2DM, or lean or normal weight with evidence of metabolic dysregulation. It is distinguished by excessive fat accumulation in hepatocytes, and a decrease in the liver's ability to oxidize fats, the accumulation of ectopic fat, and the activation of proinflammatory pathways. Chronic damage will keep this pathophysiologic cycle active causing progression from hepatic steatosis to cirrhosis and eventually, hepatocarcinoma. Epigenetics affecting gene expression without altering DNA sequence allows us to study MAFLD pathophysiology from a different perspective, in which DNA methylation processes, histone modifications, and miRNAs expression have been closely associated with MAFLD progression. However, these considerations also faced us with the circumstance that modifying those epigenetics patterns might lead to MAFLD regression. Currently, epigenetics is an area of great interest because it could provide new insights in therapeutic targets and non-invasive biomarkers. This review comprises an update on the role of epigenetic patterns, as well as innovative therapeutic targets and biomarkers in MAFLD.

## Introduction

Metabolic-associated fatty liver disease (MAFLD) is characterized by hepatic steatosis accompanied by one of three features: overweight or obesity, T2DM, or lean or normal weight with evidence of metabolic dysregulation (1).

MAFLD, as with the previous term NAFLD, represents the hepatic manifestation of a multisystem disorder, whose incidence is 20–30% in the western countries (2). Currently, there is no FDA-approved therapeutic agent for MALFD, and changes in diet and increase in physical activity are the first-in-line treatment of hepatic steatosis (3).

Gene expression is ultimately influenced by diverse epigenetic processes, including DNA methylation, histone modification, and expression of non-coding RNA molecules, like miRNAS. Epigenetic changes are reversible, and lifestyle and environmental exposure can define epigenetic patterns throughout life (4).

Epigenetic variations differ in the same individual among cell types and are associated with disease susceptibility by producing long-term changes in gene transcription (5). Alterations in hepatic epigenetics significantly contribute to MAFLD development by altering transcriptional networks implicated in redox homeostasis, peroxisome and mitochondria function, inflammation, insulin sensibility, and fat homeostasis. Most important epigenetic mechanisms implicated in the development of metabolic associated fatty liver disease are described in the next sections.

## DNA Methylation

DNA methylation is the covalent addition of a methyl group to the C5 position of cytosine generating a 5-methylcytosine (5mC), usually in cytosine–guanine dinucleotides-rich regions known as CpG islands. In general, hypermethylation of CpG islands is associated with gene repression, since the methyl group may physically block binding of transcription factors to the DNA, or it can act as a binding site for transcriptional repressors such as histone deacetylases; whereas hypomethylation is permissive to transcription (6). DNA methylation is catalyzed by a family of enzymes called DNA methyltransferases (*DNMTs*) that transfer the methyl group from an S-adenyl methionine (SAM) to DNA (7). *DNMT1* accounts for the recognition of the hemimethylated strand after a cell cycle. *DNMT3a* and *DNMT3b* are responsible for *de novo* methylation (8). The ten–eleven translocation (Tet) enzymes remove the methyl group in DNA (9).

DNA methylation is the most studied epigenetic mechanism in MAFLD. Detection of aberrant DNA methylation patterns could provide therapeutic targets and molecular tools for diagnosis and prediction of MAFLD (10). Several studies have analyzed genome-wide methylation changes associated with MAFLD, showing alterations in the methylation signature of many genes including regulatory loci for key metabolic and inflammatory pathways. For example, a study in humans using liver biopsies from obese patients with MAFLD showed methylation and expression differences in nine key enzymes implicated in intermediate metabolism and insulin signaling: pyruvate carboxylase (*PC*), ATP citratelyase (*ACLY*), phospholipase C-gamma-1 (*PLCG1*), insulin-like growth factor 1 (*IGF1*), insulin-like growth factor binding protein 2 (*IGFBP2*), and protein kinase C epsilon (*PRKCE*), putative polypeptide *N*-acetylgalactosaminyl-transferase-like protein 4 (*GALNTL4*), glutamate receptor delta-1 (*GRID1*), and inositol hexaphosphate kinase 3 (*IP6K3*) (11). A similar study founded that 41 genes responsible for lipid homeostasis were significantly and differentially methylated, including members of the APO family (lipid transport), genes involved in cholesterol transport like intracellular cholesterol transporter 1 (*NPC1L1*), acyl-CoA, sterol regulatory element binding transcription factor 1 (*SREBF1*), StAR-related lipid transfer domain containing 5 (*STARD5*), and solute carrier family 2 member 4 (*SLC2A4*) (12). Insulin resistance (IR) is part of the pathophysiology of MAFLD and its progression to NASH (13). An increased hepatic methylation of peroxisome proliferator-activated receptor gamma coactivator-1 alpha (*PPARGC1A*) has been correlated with high plasma fasting insulin levels (*r* = 0.51, *p* < 0.01) and HOMA-IR (*r* = 0.58, *p* < 0.003) in patients with MAFLD (14, 15).

## Diet and DNA Methylation

“Western diet” is characterized by excessive fat and sugar consumption and seems to contribute to MAFLD pathogenesis (16). Preclinical studies demonstrated that the consumption of high-fat diet alters DNA methylation of gene clusters (17) and induces hypermethylation in promoter regions of peroxisome proliferator-activated receptor alpha (*PPARA*) (18), whereas, high fructose induces hypermethylation of carnitine palmitoyltransferase 1A (*CTP1A*) and *PPARA* genes (19) and global hypomethylation of mitochondrial DNA (20). *PPARA* is a transcriptional regulator of genes involved in mitochondrial beta-oxidation, fatty acid transport, and hepatic production of glucose, and *PPARA* hypermethylation decreased its gene expression and induced fatty accumulation in the liver. On the other hand, peroxisome proliferator-activated receptor gamma (*PPARG*) is upregulated in diabetes, obesity, and MAFLD. Mice fed a high-fat diet (HFD) showed a reduction of the level of cytosine methylation *Pparg* promoter, DNMT activity, and induction of hepatic *Pparg* expression (21).

Furthermore, Wang et al. proposed a regulatory pathway for sugar leading to induction of lipid accumulation; Huh-7 cells administered with high-glucose showed a close relationship between an increase in nuclear 25-hydroxycholesterol and activation of *DNMT1*, which methylates cytosine of CpG in promoter regions, suppressing expression of genes involving in MAFLD diseases (22).

It is challenging to confirm these studies in humans; however, a human study examined the effect of lifestyle interventions on DNA-methylation. The participants received a regimen of either low-fat or Mediterranean-low carbohydrates for 18 months. At baseline, intrahepatic fat was inversely correlated with DNA-methylation in calcium release activated channel regulator 2A (*CRACR2A*), alpha-2-macroglobulin pseudogene 1 (*A2MP1*), and ARH/RhoGEF and pleckstrin domain protein 1 (*FARP1*) genes. In conclusion, patterns in DNA-methylation changed in *A2MP1* gene after lifestyle interventions (23).

DNA methylation patterns can be modified also by bioactive food components. For example, methyl-group donors (B9, B12, methionine, betaine, and choline) are required for SAM synthesis in one-carbon metabolism. One-carbon metabolism comprises a series of interlinking metabolic pathways that include the methionine and folate cycles that are central to cellular function, providing methyl groups for the synthesis of DNA, polyamines, amino acids, creatine, and phospholipids (24). Several studies have demonstrated that CH3 deficiency in one-carbon metabolism is strongly associated with MAFLD (25). In animal models, a deficient methyl-donor diet is associated with reduced hepatic global DNA methylation and altered DNA-methylation patterns of lipid genes associated with fatty-liver-like phenotype such as ATP binding cassette subfamily A member 1 (*Abca1*), acetyl-CoA acetyltransferase 1 (*Acat1*), 1-acylglycerol-3-phosphate O acyltransferase 3 (*Agpat3*), and angiotensin II receptor type 1 (*AGTR1*) (26, 27). In contrast, dietary methyl donor-supplementation prevents liver fat accumulation by modifying the methylation of specific gene promoters like *Srebf2, Agpat3*, and estrogen receptor 1 (*Esr1*) (28). Recently, these results were corroborated in humans; hepatic global DNA methylation levels were significantly lower in patients with MAFLD than in the control group, and also among participants who were overweight. These data correlate negatively with histological disease severity. In addition, MAFLD group had a significant higher serum homocysteine concentration (an indicator of methyl donor–deficient diet). This suggests that global DNA methylation and serum one-carbon metabolites may be markers of MAFLD status or severity (29). In patients with type 2 diabetes, a correlation between a high number of hypomethylated CpG sites and reduced levels of folate in the circulation was found (30). Another study was conducted in obese patients, associated low folate intakes with lower calcium/calmodulin-dependent protein kinase 2 (*CAMKK2*) gene methylation and IR (31).

## DNA Methylation as Predictive Biomarkers of Disease

DNA methylation in peripheral cells or ccf-DNA is a potential biomarker to diagnose MAFLD. Hypomethylation in promoters of protein kinase C epsilon (*PRKCE*) and *SEC14* like lipid binding 3 (*SEC14L3*) is associated with MAFLD by genome-wide DNA methylation profiling in peripheral blood leukocytes (32). Ma et al. reported differential methylation in 22 CpG in genes like *SLC7A11, CPT1A, SREBF1*, zinc finger RNA binding protein 2 (*ZFR2*), and *SLC9A3R1* associated with increase hepatic fat in European Ancestry participants (33). Similarly, in patients with histologically confirmed MAFLD, six differentially methylated CpG sites were identified in the Acyl-CoA synthetase long-chain family member 4 (*ACSL4*), cardiolipin synthase 1 (*CRLS1*), carnitine palmitoyltransferase 1A *(CTP1A)*, single Ig and TIR domain containing (*SIGIRR*), single-stranded DNA binding protein 1 (*SSBP1*), and zinc finger protein 622 (*ZNF622*) genes compared with healthy controls (34). Nano et al. reported an association between DNA methylation in *SLC7A11, SLC1A5, SLC43A1*, phosphoglycerate dehydrogenase (*PHGDH*), psoriasis susceptibility 1 candidate 1 (*PSORS1C1*), *SREBF1*, and ankyrin repeat and sterile alpha motif domain containing 3 (*ANKS3*) with gamma-glutamyl transferase (GGT) levels; while DNA methylation in *SLC7A11* was associated with alanine aminotransferase (ALT) (35). MAFLD may progress to advanced liver disease with the presence of fibrosis, a key histological determinant of long-term prognosis. An observational study compared liver biopsies from patients with mild vs. advanced fibrosis, identifying significant more methylation in gene regulatory regions of transforming growth factor beta 1 (*TGFB1*) and platelet-derived growth factor subunit A (*PDGFA*) in patients with mild fibrosis, whereas *PPARA* and *PPARD* showed considerably less methylation (36).

**Figure 1:**
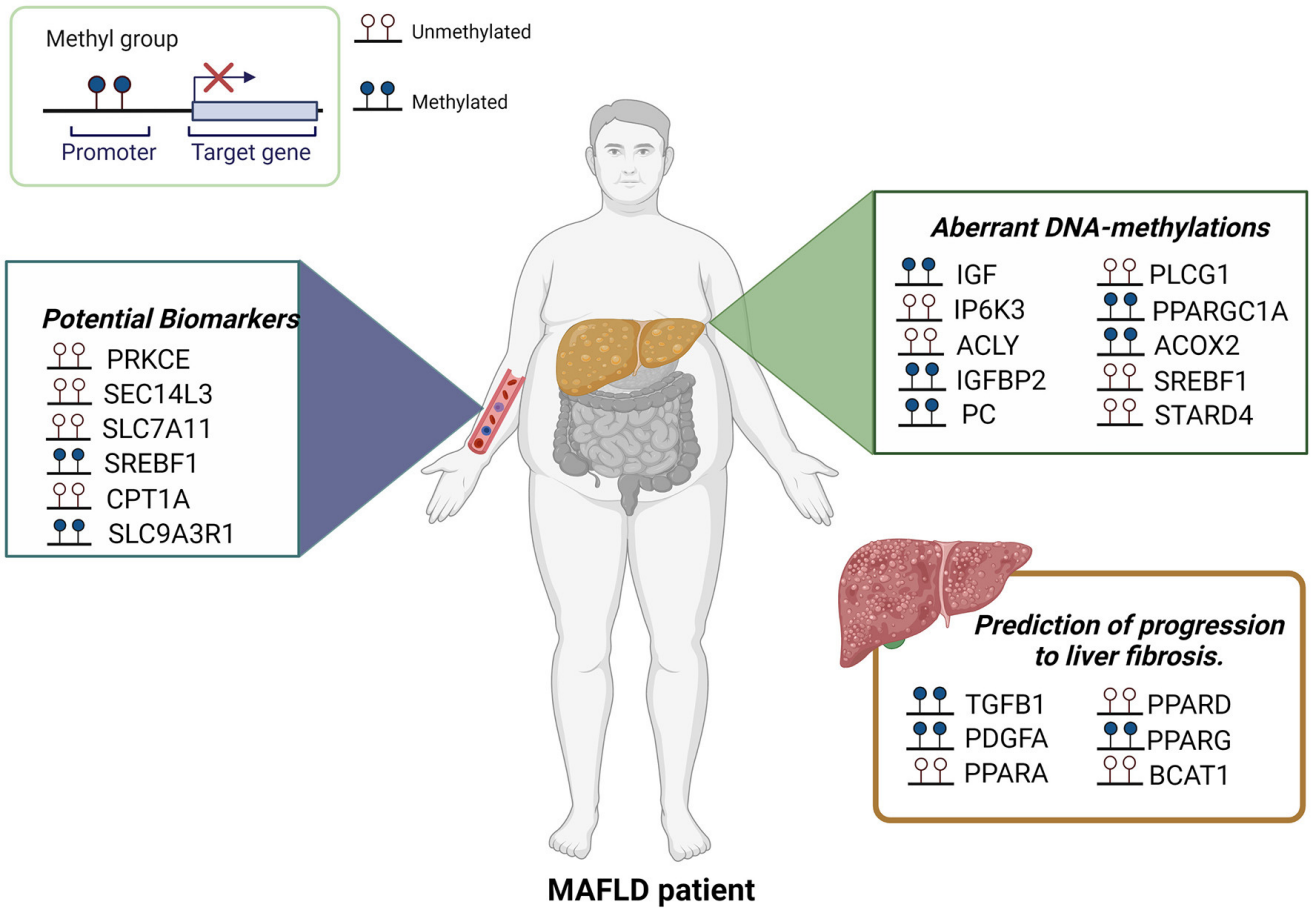
Aberrant DNA-methylations in MAFLD. Several studies using liver biopsies, ccf-DNA or peripheral blood leukocytes have been shown differential DNA methylation patterns associated with MAFLD. Certain CpGs also represent attractive biomarkers for MAFLD and the prediction of progression to fibrosis.

A previous study has demonstrated that *PPARG* promoter hypermethylation correlated with severe fibrosis in liver biopsies (37), and more recently Hardy et al. found a similar degree of hypermethylation in the *PPARG* promoter in plasma ccf-DNA and hepatocyte-rich tissue captured by laser capture microdissection, suggesting that plasma DNA methylation of *PPARG* could potentially be used as a noninvasive method to determinate liver fibrosis severity in patients with MAFLD (38). Also, hypomethylation in a branched chain amino acid transaminase 1 (*BCAT1*) has been reported inversely associated with fibrosis degree (39). Hypomethylation of fibroblast growth factor receptor 2 (*FGFR2*), caspase 1 (*CASP1*), and hypermethylation of methionine adenosyltransferase 1A (*MAT1A*) were associated with advanced MAFLD in a study of Murphy et al. (40). Parvin beta variant 1 (*PARVB*) (hypomethylated in CpG26) and patatin like phospholipase domain containing (*PNPLA3*) (hypermethylated in CpG99) have also been associated with MAFLD (41). Figure 1 describes differential DNA methylation patterns associated with MAFLD, some of them proposed as biomarkers.

## Histone Modifications in MAFLD

Histones are a family of basic proteins whose positive charges allow them to associate with DNA in the nucleus and help them condense it into a chromatin. The basic structural unit of chromatin, the nucleosome (42), is formed by a pair of each H2a, H2b, H3, and H4 histones, an octamer (43). These histones are small globular proteins containing an N-terminal tail that can undergo acetylation, methylation, phosphorylation, SUMOylation, ubiquitination, or ADP-ribosylation. Multiple histones modifying enzymes can carry out more than 60 chemical histone-modifications that affect specific DNA binding sites, causing transcription activation or silencing of specific genes (44).

Lysine acetylation or methylation in the N-terminal tail stands out as the histone modifications with greatest repercussion in gene expression (45). Acetylation is mediated by histone acetyltransferases (HAT) and is usually associated with active gene transcription due to its ability to decompress chromatin. For this reaction, acetyl CoA acts as a cofactor, and subsequently HAT catalyzes the transfer of an acetyl group to the epsilon-amino group of lysine (46), neutralizing the positive charge of lysine and weakening histone and DNA interactions (47). In the opposite way, histone deacetylases (HDAC) remove acetyl groups from lysine and thus restores the compacted form of chromatin (48).

**Figure 2:**
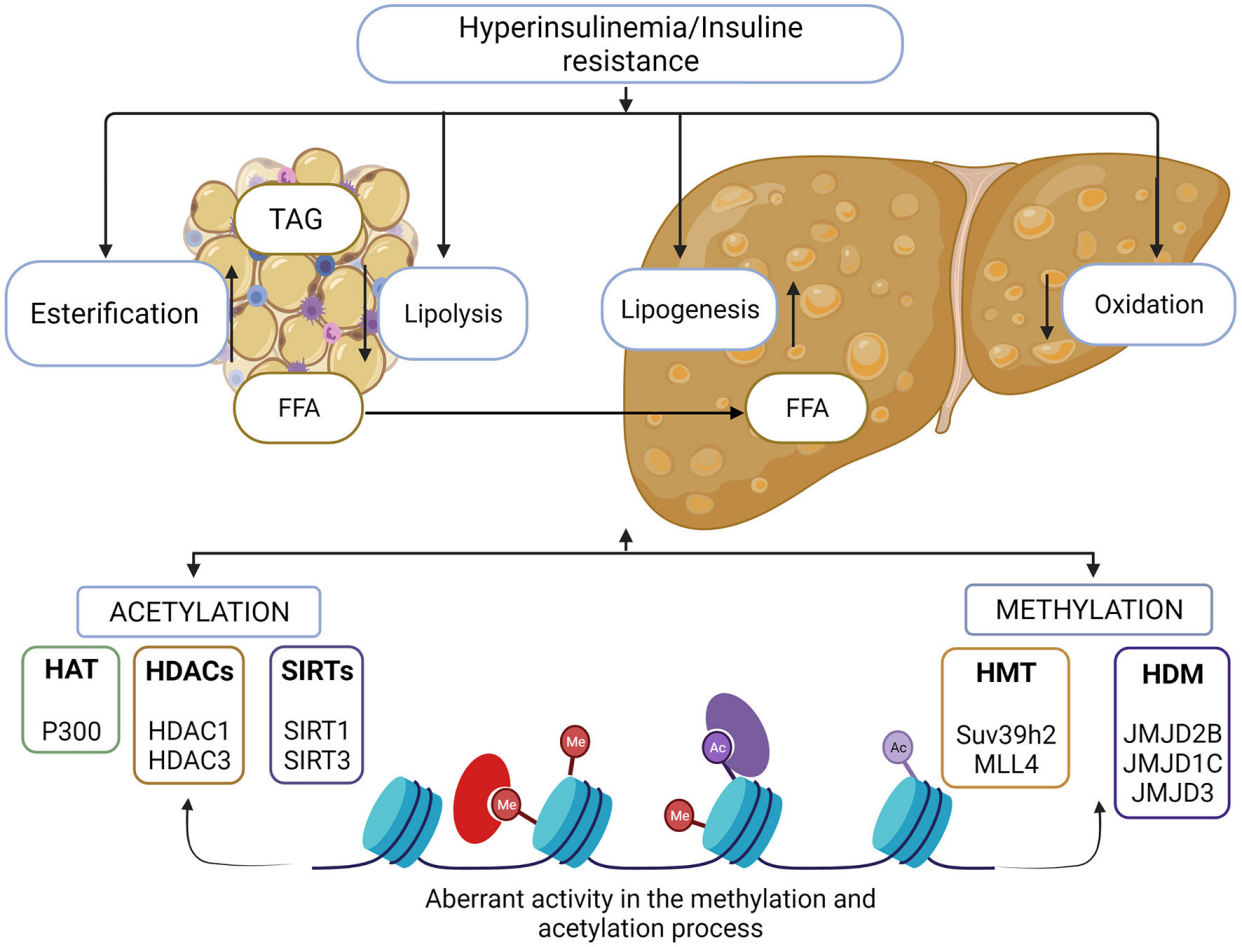
Histone modifications in MAFLD. Metabolic imbalance in lipids and carbohydrates are involved in the development of MAFLD. Adipose tissue storage and insulin resistance triggers the accumulation of free fatty acids in the liver and liver homeostasis is lost. There is a direct association between aberrant chromatin modifications and this metabolic imbalance. Histone methylation and acetylation process, allows the activation of genes associated with the lipogenic and inflammatory process, as well as the reduction in expression of the genes involved in the oxidation of fatty acids, enzymes responsible for these events are possible therapeutic target for MAFLD control.

On the other hand, histone methylation in residues in the N-terminal tail of histones causes silencing of chromatin and the inactivation of transcription. However, in particular cases, methylation of histone activates gene transcription and is associated with the initiation of chromatin remodeling (49). The precise effect of methylation is linked to the specific residue where the reaction takes place. The methylation process is carried out by histone methyltransferases (HTM), which have the ability to add one, two, or three methyl groups to lysine or arginine residues of histones. Histone demethylases (HDM) have the ability to remove methyl groups from histone, thus beginning the remodeling of chromatin toward a decompressed or active state. HDMs have been classified into two classes, the FAD-dependent amino oxidases (LSD) and the jumonji C demethylase (JMJD) (50). Imbalance in histone modifications causes a disproportion in transcriptional activity associated with the development of diseases such as type 2 diabetes mellitus, obesity, and consequently MAFLD (51). Main enzymes involved in histone modifications that are implicated in MAFLD development are enlisted in Figure 2.

## Histone Acetylation

During IR or DM2 the risk to develop non-alcoholic fatty liver increases due to inflammatory factors; where nuclear factor enhancing kappa of activated B cells (*NFkB*) or elements of carbohydrate metabolism that affect lipogenesis like element binding protein carbohydrate response (*ChREBP*) stand out. These factors are upregulated by some HAT (52). P300, a member of the HAT family, is a transcriptional regulator that plays a very important role modifying *NFkB* pathway. It has been shown that inhibition of p300 improves MAFLD in mice and restores biochemical parameters, decreases activity of genes involved in lipogenesis, and therefore, the aberrant activity of p300 favors MAFLD development (53). One of the main factors that is altered by p300 is *ChREBP*, a protein essential for the accumulation of fat in the liver. Bricambert et al. corroborated the interaction of these two molecules, activating or inhibiting kinase inducible by serine/threonine kinase 2 (*SIK2*), an element that regulates the activity of p300. In HepG2 cells and mice, SIK2 inhibited p300 activity by direct phosphorylation, and therefore also decreased the lipogenesis mediated by *ChREBP*. *SIK2* depletion caused an overexpression of p300 increasing lipogenesis and causing insulin resistance, hepatic steatosis and inflammation (54). HDACs have 4 families (class I, IIa, IIb, and IV) that differ in structure, enzymatic function, and location. HDACs play an important role in the development of MAFLD, some with more evidence than others. For example, *HDAC1*, a member of the class I family-depleted HepG2 cells decreased sterol regulatory element binding protein (*SREBP1c*) as well as, liver tissue of P50 *NFkB*-subunit KO mice (55). In addition to *HDAC1*, the activity of *HDAC3* has also been evaluated in MAFLD and in obesity and insulin resistance. *HDAC3* regulates hepatic lipid metabolism in the opposite way to *HDAC1*. *HDAC3* is an important lipid homeostatic regulator in the liver, and its loss leads to severe hepatic steatosis in mice (56). It is key to highlight that *HDAC3* also has direct interaction with molecules that participate in the development of hepatic steatosis, such as *SREBP1*, key molecule in the lipogenic process (57). In addition, *HDAC3* has a specific role in the circadian pattern of hepatic lipogenesis, a dysregulation in this cycle mediated by *SREBP1* increases the lipogenic process (58, 59).

A well-known group of deacetylases are silent information regulatory proteins (SIRTs), also known as Sirtuins. SIRTs are members of the class III HDAC family and use NAD^+^ as a cofactor. They can interact with histones as well as non-histone proteins and have gained interest in metabolic diseases since they are involved in lipid homeostasis, oxidative stress, and insulin resistance, all events implicated in MAFLD development. Sirtuin family has 7 members characterized by their structure, enzymatic function, and localization. *SIRT 1, 2, 3, 6*, and *7* are mainly found in the nucleus, *SIRT1* and *SIRT2* are also in the cytoplasm, and *SIRT4* and *SIRT5* in mitochondria (60).

The sirtuins with greatest association to nonalcoholic fatty liver disease development are *SIRT1* and *SIRT3*. Recent evidence showed that SIRT1 is an important piece in lipid homeostasis in the liver, and it is an agonist ligand of peroxisome proliferator-activated receptor alpha (*PPAR*), promoting oxidative activity in lipids. Sandoval-Rodriguez and Monroy-Ramirez et al. used synthetic inhibitors and activators of *SIRT1* and *PPARA* in cultured HepG2 cells, demonstrating positive feedback between both proteins, which leads to the fact that a decrease in *SIRT1* favors the development of MAFLD in part due to decrease in *PPARA* activity (61). The effect of *SIRT1* on lipid metabolism has been an important part of the discussion of whether it could function as a therapeutic target for MAFLD. The activation of *SIRT1* during MALFD decreases lipids and TGs accumulation in the liver, decreasing inflammation and lipogenic process (62). For its part, *SIRT3* is also important in MAFLD. Mice deficient in *SIRT3* and fed with HFD increased lipid levels in the liver, promoting development of MAFLD. *SIRT3* deficiency leads to less DNA binding activity in *PPARA*, thereby decreasing the production of molecules activated by *PPARA*; promoting fatty acids oxidative status (63). In addition, regarding oxidative stress and mitochondrial damage, events involved in hepatic steatosis, *SIRT3* deficiency increased oxidative stress and activation of caspase-9 pathway. However, overexpression of *SIRT3* decreases reactive oxygen species and promotes the activation of the *ERK-CREB-Bnip3* pathway improving mitophagy (63).

## Histone Methylation

Transcription silencing is linked with a compacted state of chromatin, generally, associated with methylation of histone tail. Histone 3 lysine 9 (*H3K9*) has been associated with the development of MAFLD, and the aberrant activity of some methyltransferases have been associated with this process (51).

Histone–lysine *N*-methyltransferase SUV39H2, an enzyme capable of adding mono, di, and trimethylated labels to H3K9, has a fundamental role in the activation of inflammatory pathways. Also, it can reduce the activity of SIRT1 causing NASH progress. SUV39H2 activity was analyzed in KO mice fed a HFD, and they developed hepatic steatosis of less severity compared with the wild type for this enzyme (64).

In addition, there is also a relationship between the development of hepatic steatosis and methylation of histone 3 lysine 4 (*H3K4*) by myeloid/lymphoid or mixed-lineage leukemia 4 (*MLL4*) methyltransferase. It was shown that in overnutrition conditions, *MLL4* provokes *H3K4* methylation facilitating interaction with targets of peroxisome proliferator-activated receptor gamma 2 (*PPARy2*), which promotes lipogenesis (65).

On the other hand, the activity of methylases has also been studied in MAFLD. Clear evidence of the direct effect of *JMJD2B* on histone mark H3K9 has demonstrated the importance of this enzyme in the lipogenic process during MAFLD, with the interaction of *PPARG2* and the ligand-activated liver X receptor alpha (*LXRa*). JMJD2B removes the trimethylated and dimethylated marks, leaving the monomethylated mark of H3K9, causing activation of *PPARG2* and its target genes increasing the hepatic lipogenic process (66). The same situation occurs with Liver X receptor alpha (*LXRA*). It has been shown that the overexpression of JMJD2B increases the activity of this receptor, inducing intracellular accumulation of triglycerides and thus MAFLD development (67).

Another molecule that has a demethylase function and that has been associated with the progression of MAFLD is JMJD1C. In the same way, the interaction of this enzyme with the histone mark H3K9, removing repressive marks, promotes the transcription of genes, inducing lipogenesis and accumulation of hepatic fatty acids. It has been shown that the mammalian Target of Rapamycin (mTOR) complex phosphorylates JMJD1C, allowing interaction with upstream stimulatory factor 1 (USF1), a molecule that activates lipogenic genes and is associated with familial hyperlipidemia (68).

On the contrary, the activity of JMJD3 has been associated with the disease improvement; it removes the repressive mark of histone 3 lysine 27 (H3K27) leaving it in its dimethylated form (H3K27me2), promoting chromatin remodeling, and in turn, working together with *SIRT1*, to promote *PPARA* activation. This evidence was obtained in fasting conditions, and genes involved in gluconeogenesis pathway had no relevant activity, but these facts open up the possibility of a new therapeutic target (69).

## microRNAs

**Figure 3:**
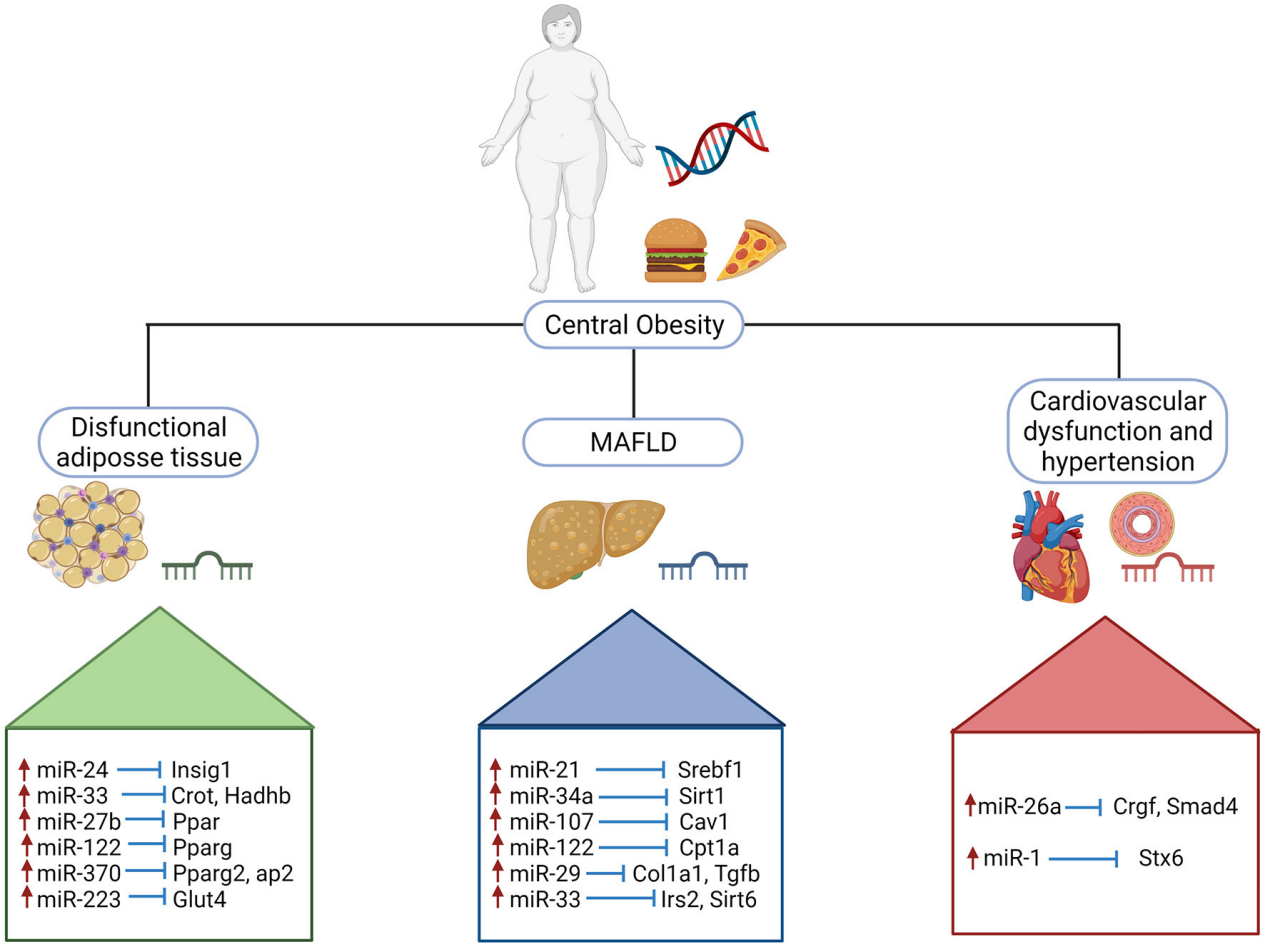
Upregulated miRNAs involved in pathogenesis and development of MAFLD. Schematic illustration of selected miRNAs shows the impact on the stimulatory effect on target genes implicated in obesity, metabolic dysregulation and cardiovascular dysfunction. Insig 1, insulin induced gene 1; Crot, carnitine O-octanoyltransferase; Hadhb, hydroxyacyl-coa dehydrogenase trifunctional multienzyme complex subunit beta; Ppar, peroxisome proliferator activated receptor; Pparg, peroxisome proliferator activated receptor gamma; Pparg2, peroxisome proliferator activated receptor gamma 2; aP2, activating protein 2; Glut4, glucose transporter type 4; Srebf1, sterol regulatory element binding transcription factor; Sirt1; sirtuin 1; Cav1, caveolin 1; Cpt1a, carnitine palmitoyltransferase 1A; Col1a1, collagen type I alpha 1 chain; Tgfb, transforming growth factor beta; Irs2, insulin receptor substrate 2; Sirt6, sirtuin 6; Crgf, teratocarcinoma-derived growth factor 1; Smad4, SMAD Family Member 4; Stx6, syntaxin 6.

microRNAs (miRNAs) are single-stranded non-coding RNAs of 18–25 nucleotides long that can regulate gene expression at posttranscriptional level by inhibiting translation or inducing degradation of target mRNAs through complementary base-pairing (70). miRNAs account for 1–5% of the human genome and regulate at least 50% of protein coding genes in mammals (71). To date, more than 2,800 human miRNAs have been registered in the miRBase 22.1, which are predicted to regulate up to 60% of the human genes. About 50% of miRNAs are transcribed from protein coding genes, mostly intragenic regions particularly introns and few exons. The other half are intergenic, transcribed independently, and regulated by their own promoters. Each miRNA can regulate several target genes, and vice versa, and each target gene can be regulated by various miRNAs, explaining why miRNAs can play crucial functions in essentially all biological processes and in all cell types (72). Evidence have demonstrated that miRNAs are implicated as important mediators in metabolic diseases including obesity, DM2, metabolic syndrome, and metabolic associated fatty liver disease (MAFLD) (73–75). Figure 3 summarizes upregulated miRNAs involved in pathogenesis and development of MAFLD.

## miRNAs in Obesity

Several miRNAs including miR-27b, miR-33, miR-34a, miR-122, and miR-223 are important regulators in fatty acid metabolism and cholesterol biosynthesis in the liver (76). Specifically, miR-33 plays a key role in cholesterol homeostasis thought suppression of sterol regulatory element-binding protein 1 (*SREBP1*), high density lipoprotein formation, fatty acid oxidation, and insulin signaling (77).

miR-27b-3p exert regulatory effects in lipid metabolism and is altered in dyslipidemia (78). In high-fat diet model of obesity, miR-27b-3p suppress adipose tissue browning. Due to this key role in promoting body fat accumulation miR-27b-3p should be further explored as a potential target for the treatment of central obesity and linked diseases (79).

miR-122 is the most abundant miRNA in the liver, and has a key role in liver metabolism, cholesterol biosynthesis, fatty acid synthesis, and oxidation (80). It should be noted that miR-122 was the first miRNA to be associated with metabolic regulation (81). Long JK et al. found that miR-122 promoted hepatic lipogenesis inhibiting *LKB1*/*AMPK* pathway by targeting *SIRT1* in HepG2 and Huh-7 cells cultured with free fatty acids (FFA) (82). miR-122 was downregulated in steatotic-FFA-induced hepatocytes, and nonalcoholic steatohepatitis mice model using streptozotocin and HFD (STZ- HFD). Besides, miR-122 showed an important role in hepatic triglyceride accumulation reducing *YY1* mRNA stability causing upregulation in *FXR-SHP* signaling (83).

miR-34a has been reported as a probable tumor suppressor in numerous types of cancers (84). miR-34a is upregulated in MAFLD and is an essential regulator of lipid metabolism (85). In a work by Ding et al., miR-34a levels were increased in L02 cells transfected with miR-34a inhibitor and C57BL/6 mice injected with a miR-34a inhibitor. *Ppara* and *Sirt1*, which are target genes of miR-34a, were downregulated after miR-34a inhibitory treatment, provoking triacylglycerides, liver index, and activated-AMPK pathway decrease (86). In adipose tissue it has been reported that miR-34a expression gradually increases as dietary obesity develops. In miR-34a–KO mice glucose intolerance, insulin resistance, and systemic inflammation were present in epidydimal white adipose tissue (epiWAT).

Interestingly, increased miR-34a expression causes adipose inflammation principally by reduced expression of *Klf4*, resulting in suppressive effects on M2 macrophages polarization. Besides, it was found that high expression of miR-34a in visceral fat of overweight/obese patients correlated negatively with diminished *Klf4* (87).

miR-33 is a key regulator of lipid metabolism by targeting genes involved in cholesterol uptake and efflux in the liver, fatty acid metabolism *Cpt1, Crot, Hadhb*, insulin signaling *IRS2* and mitochondrial function *Ampk, Pgc1a* (88–90). miR-223 could inhibit cholesterol biosynthesis in mice through negative regulation of the 3-hydroxy-3-methylglutaryl-CoA synthase 1 (*Hmgcs1*) and the sterol-C4-methyloxidase-like protein (*Sc4mol*). Besides, miR-223 decreased high-density lipoprotein-cholesterol (HDL-C) uptake by targeting the scavenger receptor class B member 1 causing *ABCA1* expression increase that rise cholesterol efflux (91). Otherwise, miR-223 targets include inflammatory and oncogenic genes like *CXCL10* and *TAZ*, data obtained in hepatocytes of high fat diet fed mice and in NASH patient livers. Therefore, miR-223 could protect against NASH development 322 (92).

A recent study by Zhang et al. reported that overexpression of miR-802 downregulates insulin transcription and secretion, as well as impairs glucose tolerance, suggesting a role of miR-802 in the development of obesity-associated β cell dysfunction (93). Several studies have reported that miR-221 is upregulated in adipose tissue from obese patients (94, 95). Peng et al. suggested that miR-221 promotes white adipose tissue inflammation and reduces insulin sensitivity in obesity while suppressing *SIRT1* (96).

## miRNAs in Metabolic Alterations

Some miRNAs are crucial in MAFLD progression and metabolic alterations including waist circumference, blood pressure, serum triglycerides, and HOMA levels (97).

During adipogenesis, miR-425 expression is controlled by *Pparg* in adipocytes. miR-425 overexpression resulted in a proliferation reduction of 3T3-L1 preadipocytes, but accelerated cellular adipogenic differentiation. miR-425 also influences adipogenesis inhibiting its target gene *Mapk14*,a negative regulator of adipogenesis (98).

miR-107 is known to regulate insulin sensitivity in mouse models mainly by altering liver metabolism. miR-107 has a key role in lipid metabolism, inhibiting *CDK6* expression and its downstream targets, reducing adipogenesis in preadipocytes. Besides, it has been proposed that miR-107 promotes ectopic fatty acid accumulation and reduced glucose tolerance since miR-107 decreased glucose uptake and triglycerides synthesis in mature adipocytes (99). In a work carried out by Okamoto et al., serum miR-379 expression was upregulated in patients with MAFLD compared with healthy individuals. Serum levels of miR-379 showed positive correlations with alkaline phosphatase, total cholesterol, low- density-lipoprotein cholesterol, and non-high-density lipoprotein cholesterol levels in patients with early stage MAFLD (100).

miR-126a has been validated as a biomarker in obesity and related metabolic disease in women by Vonhögen et al. Thus, circulating levels of miR-216a are predictive factors for obesity. Interestingly, they found the obesity predisposition locus, the miR-216a gene that includes CpG islands with differential DNA methylation levels among obese and non-obese children, and is related with differential circulating miR-216a plasma levels in obese and non-obese women (101).

Remarkably, Lin et al. demonstrated that miR-144 targets *Foxo1*, thus reducing its expression and inhibiting its promotional effect on adiponectin, thereby alleviating the inhibitory effect of adiponectin on adipogenesis in an experimental model (102). A study performed by Komaya et al. reported that miR-33b showed high expression in the liver, and its expression was increased in response to cholesterol overload, using genetically modified mice, miR-33 knockout mice, and miR-33b Knock in mice; as a result, miR-33b showed increased atherogenic potential (103).

Basic and clinical evidence has shed light on the association between MAFLD and cardiovascular diseases (CVD) (104); in this context, increased plasma miR-1 was found to be associated with myocardial steatosis and it has been suggested to be a biomarker for diabetic cardiomyopathy (105).

A recent work carried out by Jiang et al. reported that miR-1 expression was increased in liver tissues and primary hepatocytes derived from a diet-induced obese mice, as well as, selective increase of miR-1 expression in EVs derived from steatotic hepatocytes (106). Several studies have shown that miR-26a is highly associated to cardiovascular diseases. Zhang et al. reported that miR-26a prevented blood pressure elevation and inhibited myocardial fibrosis using hypertensive animal models (107). Figure 3 schematizes crucial miRNAs involved in pathogenesis and development of MAFLD, considering key parameters such as obesity, type 2 diabetes mellitus, and metabolic alterations (hypertension, high level of triglycerides and cholesterol, and HOMA). Table 1 lists the miRNAs implicated in crucial key process in development of MAFLD and their potential target genes.

**Table 1:** miRNAs implicated in crucial key process in MAFLD and their potential target genes.

**miRNAS**	**Expression in MAFLD**	**Potential target genes**	**References**
miR-24	Upregulated	*Insig1, Srb1*	(108, 109)
miR-33a/b	Upregulated	*Crot, Hadhb, Irs2, Sirt6, Dusp1, Tfrc, Abca1, Ski, Hipk2*	(103, 110)
miR-27b	Upregulated	*Ppar, Acot2*	(111)
miR-192	Downregulated	*Scd1*	(82)
miR-122	Upregulated	*Pparg, Agpat1, Dgat1, Cpeb1,Sirt1*	(112, 113)
miR-144	Upregulated	*Abca1*	(114)
miR-148a	Upregulated	*Ldlr, Pgc1a, Insig1*	(115)
miR-223	Upregulated	*Glut4, Nlrp3, Igf1r, Cxcl10*	(116)
miR-145	Downregulated	*Klf4*	(117)
miR-21a	Upregulated	*Srebf1, Smad7, Ppara*	(118, 119)
miR-107	Upregulated	*Cav1, Srebf1, Cpt1a*	(120)
miR-34a	Upregulated	*Sirt1, Hnf4a, Ppara*	(86, 121)
miR-29	Upregulated	*Col1a1, Tgfb, Sirt1*	(122)
miR-26a	Upregulated	*Crgf, Smad4, Eif2a*	(123)
miR-1	Upregulated	*Stx6*	(124)

*Insig1, insulin induced gene 1; Srb1, scavenger receptor class B type 1; Crot, carnitine O-octanoyltransferase; Hadhb, hydroxyacyl-coA dehydrogenase trifunctional multienzyme complex subunit beta; Irs2, insulin receptor substrate 2; Sirt6, sirtuin 6; Dusp1, dual specificity phosphatase 1; Tfrc, transferrin receptor; Abca1, ATP binding cassette subfamily A member 1; Ski, SKI Proto-Oncogene; Hipk2, homeodomain interacting protein kinase 2; Ppar, peroxisome proliferator activated receptor; Acot2, acyl-CoA thioesterase 2; Scd1, stearoyl-CoA desaturase 1; Pparg, peroxisome proliferator activated receptor gamma; Agpat1, 1-acylglycerol-3-phosphate O-acyltransferase 1; Dgat1, Diacylglycerol O-Acyltransferase 1; Cpeb1, cytoplasmic polyadenylation element binding protein 1; Sirt1, sirtuin 1; Glut4, glucose transporter type 4; Nlrp3, NLR family pyrin domain containing 3; Igf1r, insulin like growth factor 1 receptor; Cxcl10, C-X-C motif chemokine ligand 10; Smad7, SMAD Family Member 7; Klf4, Kruppel Like Factor 4; Ppara, peroxisome proliferator activated receptor alpha; Cav1, caveolin 1; Srebf1, sterol regulatory element binding transcription factor 1; Cpt1a, carnitine Palmitoyltransferase 1A; Hnf4a, Hepatocyte Nuclear Factor 4 Alpha; Col1a1, collagen Type I Alpha 1 Chain, Tgfb, transforming Growth Factor Beta 1; Crgf: teratocarcinoma-derived growth factor 1; Smad 4: SMAD Family Member 4; Eif2a, eukaryotic Translation Initiation Factor 2A; Stx6: syntaxin 6*.

## Clinical Trials Involving miRNAs for Hepatic Diseases

In the last decade, various miRNA-based therapeutics have been tested in different clinical trials. The first anti-miRNA drug for the treatment of hepatitis C is a locked nucleic acid (LNA) that inhibits miR-122, called Miravisen. Miravirsen inhibits miR-122 biogenesis and repressed HCV infection. miR-122 has a critical role in the life cycle of HCV due to the fact that miR-122 binds to two target sites (S1and S2) at the 5' end of the HCV genome, forming an oligomeric miR-122–HCV complex that protects the HCV genome from nucleolytic degradation or from host innate immune responses. Besides, at least three additional target sites in 3'-untranslated region of HCV genome have not been of functional importance (125). Currently, anti-miR-122 safety and effectiveness is being evaluated in a phase II clinical trial (126).

RG-101 is another novel anti-miR 122 for the treatment of hepatitis C virus. It is an N-acetylgalactosamine (GalNAc)-conjugated oligonucleotide. RG-101 repressed replication of HCV genotypes 1a and 1b in replicon systems. However, the precise mechanism of HCV suppression by RG-101 is not yet identified (127). Currently, RG-101 has reached the phase 1B clinical trial (128, 129).

Another miRNA-based therapeutic, a GalNAc conjugated anti-miR 103/107, called RG-125 (AZD4076) is an insulin sensitizer to treat patients with metabolic diseases such as type 2 diabetes and NASH. It has been reported that RG-125 normalized glucose tolerance and improved HOMA-IR in obese-diet induced mice compared with the control group. RG-125 treatment also reversed the extreme hyperglycemia that develops with age in db/db mice (130). Table 2 lists the clinical trials using miRNAs-based drugs registered in the ClinicalTrials.gov web site (August 2021) for liver diseases.

**Table 2:** Clinical trials using miRNAs for hepatic diseases.

**Start year**	**miRNA source/type**	**Study type**	**Characteristics**	**Status**	**ClinicalTrials identifier**	**Authors**
2021	Panel of circulating miRNAs (not specific)	Observational cohort prospective	Early detection of hepatocellular carcinoma (HCC): miRNA, microbiome and imaging biomarkers in the evolution of chronic liver disease in a high-risk	Recruiting	NCT04965259	Pierce Chow, et al.
2020	Serum circulating miRNAs	Observational cohort prospective	Hepatic microRNA expression in non alcoholic fatty liver disease	Not yet recruiting	NCT04574557	Nourhan M.Abbas, et al.
2019	miRNA profile (not specific)	Observational case-only prospective	Expression and variance of microRNAs in a cohort of patients with acute decompensation of cirrhosis	Recruiting	NCT03905746	Fanny Lebossé et al.
2017	Serum circulating miRNAs miR-122-5p, miR-126a-3p, miR-193a-5p, miR-222-3p	Interventional clinical trial randomized parallel assignment	Effects of a combination of prebiotic fibers on weight loss during an energy restricted diet in an overweight/obese population	Completed	NCT03135041	Thomas M. Larsen et al.
2016	Plasma circulating miRNA panel	Observational prospective cohort	Comparative study of circulating microRNA changes in patients with liver injury and healthy subjects	Recruiting	NCT03000621	Huang Jian et al.
2016	anti-miR-103/107 (RG-125)	Interventional clinical trial randomized parallel assignment single masking	Study to assess the safety, tolerability, pharmacokinetics and pharmacodynamics of AZD4076 following multiple ascending dose administration to T2DM Subjects with NAFLD	Completed recruiment	NCT02826525	Linda Morrow et al.
2015	Serum miRNAs	Interventional randomized parallel assignment	Impact of IL-28B rs12979860 and rs4803217 gene polymorphisms associated with miRNAs deregulation on HCV-related hepatocellular carcinoma	Not yet recruiting	NCT02507882	Waleed Samir, et al.
2013	Liposomal injection of miR-34a mimic	Interventional clinical trial single group assignment open label	A multicenter phase I study of MRX34, MicroRNA miR-RX34 Liposomal Injection	Completed five immune related serious adverse events	NCT01829971	O'Neill Vincent, et al.
2010	antimiR-122 (Miravirsen)	Interventional clinical trial randomized parallel assignment Double masking	Multiple ascending dose study of miravirsen in treatment-naïve Chronic Hepatitis C subjects	Phase II	NCT01200420	Zeuzem et al.

## Conclusion and Perspectives

Metabolic dysfunction associated fatty liver disease is currently a global health problem, epidemically associated to obesity, metabolic syndrome, and type II diabetes mellitus. MAFLD development and progression involves several genetic and environmental factors including epigenetics. Epigenetics includes an extensive amount of events such as methylation in CpGs, chemical modification of histones, and posttranscriptional gene regulation by the modification of mRNA stability through short noncoding RNAs such as miRNAs. In latest years, epigenetic modifications in DNA and histone have been studied as essential mechanisms that modify the development of liver diseases including MAFLD. Hence the dysregulation of epigenetic modifications has a critical role in MAFLD progression since it regulates the expression and activity of various genes implicated in lipid metabolism, insulin resistance, DNA repair, and inflammatory process that enhance the pathogenesis of MAFLD (10, 131). Currently, it has been demonstrated that miRNAs involved in lipid synthesis, fatty acid, and glucose catabolism and inflammation are dysregulated in MAFLD being useful as biomarkers (132). Moreover, it has been suggested that precise methylation patterns in DNA may be used as a predictor or diagnostic for MAFLD progression (133). Besides, the crucial paper of numerous micronutrients seems necessary to maintain DNA methylation homeostasis, as they act as cofactors of a variety of enzymes involved in DNA methylation, synthesis, and repair (134). To date, no therapeutic strategy is approved for the treatment of MAFLD, and lifestyle modifications, physical exercise, and weight loss account as the keystone therapeutics for patients with MAFLD. Certainly, a profound understanding of the molecular mechanisms related to gene expression, epigenetic modifications, and environment interactions ought to be a main concern for future studies. Overall, further basic research is necessary to improve mechanistic knowledge of the epigenetic processes and their interactions, their dysregulation in MAFLD, and the molecular and cellular response to epigenetic-based therapies. These studies together with clinical trials will enhance epigenetic-based personalized medicine. In conclusion, research in this area is in constant advance; however, there is still more to study to increase our understanding in MAFLD.

## Author Contributions

JR-S and RE-G contributed to planning, bibliographic revision, writing of the manuscript, and figures design. RR-C contributed to the writing of the manuscript and literature review. JA-B contributed to figures design and writing and revising of the manuscript. AS-R was responsible for the manuscript planning and revising. All authors have read and agreed to the published version of the manuscript.

## Funding

This work was supported by the Fondo de Desarrollo Científico de Jalisco (FODECIJAL) Grant 7941-2019 awarded to JA-B.

## Conflict of Interest

JA-B is a consultant for CellPharma Inc. The remaining authors declare that the research was conducted in the absence of any commercial or financial relationships that could be construed as a potential conflict of interest.

## Publisher's Note

All claims expressed in this article are solely those of the authors and do not necessarily represent those of their affiliated organizations, or those of the publisher, the editors and the reviewers. Any product that may be evaluated in this article, or claim that may be made by its manufacturer, is not guaranteed or endorsed by the publisher.
